# Atypical reproductive cycles in a population of *Sceloporus grammicus* (Squamata: Phrynosomatidae) from the Mexican Plateau

**DOI:** 10.1002/ece3.310

**Published:** 2012-07-06

**Authors:** Aurelio Ramírez-Bautista, Barry P Stephenson, Abraham Lozano, Héctor Uribe-Rodríguez, Adrian Leyte Manrique

**Affiliations:** 1 Centro de Investigaciones Biológicas, Universidad Autónoma del Estado de HidalgoA.P. 1-69 Plaza Juárez, C.P. 42001, Pachuca, Hidalgo, México; 2 Department of Biology, Mercer University1400 Coleman Ave., Macon, Georgia, 31207; 3 Estación Biológica, Instituto Tecnológico Superior de IrapuatoCarretera Irapuato-Silao km 12.5, s/n, Col. El Copal, C.P. 36821, Irapuato, Guanajuato, México

**Keywords:** Interpopulation variation, life history, lizard, reproduction, sexual dimorphism

## Abstract

The spiny lizard *Sceloporus grammicus* (Squamata: Phrynosomatidae) is a small reptile from central México and the southern United States, occurring in a wide geographic area characterized by extensive variation in topographic and climatic regimes. Genetic variation among lineages from central México is substantial, though the extent to which this variation corresponds with life-history traits remains obscure. To address part of this puzzle, we studied a population of *S. grammicus* from Tepeapulco, Hidalgo, México. Male-biased sexual dimorphism was extensive in this population; males were larger than females overall, and expressed proportionately larger heads and longer limbs. Minimum size at sexual maturity was similar in the sexes (males: 43 mm; females: 42 mm). In contrast to other populations from the Central Plateau, reproductive activity of males and females was synchronous. Testicular recrudescence of adult males was initiated in October–November, and maximum testis size maintained from December to July. Female reproductive activity showed no clear seasonal pattern: females had vitellogenic follicles from October to July, and pregnant females were found throughout the year. Female body size was not related to litter size. Neither male nor female gonadal mass was correlated with any abiotic environmental variable examined. Differences in reproductive characteristics among populations of *S. grammicus* might be indicative of plasticity in response to local environmental conditions, local adaptation, or complex gene × environment interactions. We consider these results in the context of previously studied populations of *S. grammicus* from the Central Plateau and elsewhere, and propose directions for future research.

## Introduction

An important area of research in animal ecology aims to understand the extent to which intraspecific variation in traits associated with reproductive phenology has primarily an environmental or genetic basis. When variation in reproductive activity and related life-history traits is largely (if not exclusively) a function of phenotypic plasticity, the effects of such variation on local population adaptation should be small. Conversely, heritable variation for reproductive traits can potentially generate important effects on fitness. These effects should manifest themselves in local adaption that may ultimately lead to the evolution of new species.

Intraspecific studies of reproductive patterns in lizards have been an important source of investigation in this area. Such studies have revealed geographic variation in a variety of life-history characteristics, such as litter size, egg size, fecundity, and age at sexual maturity among populations (Michaud and Echternacht 1995). The genus *Sceloporus* (Squamata: Phrynosomatidae) in particular has proven to be an excellent model system for addressing questions related to the role of the environment and life-history traits (Guillette and Méndez-de la Cruz 1993; Méndez-de la Cruz et al. 1998; Hernández-Salinas et al. 2010) such as length of reproductive season, litter size, gestation time, and body size at sexual maturity (e.g., *S. jarrovii*: Goldberg 1971; *S. variabilis*: Benabib 1994). Variation in these traits has been suggested to be related to food availability (Ballinger 1977; Dunham 1982), duration of appropriate environmental conditions (Benabib 1994), or phylogenetic history (Dunham and Miles 1985).

The *Sceloporus grammicus* group consists of six to eight species of spiny lizard primarily distributed in northern and central México (Lara-Góngora 2004; Wiens et al. 2010). Most species in the group are restricted to relatively small areas in central México (Arévalo et al. 1991). For example, *S. heterolepis* and *S. shannonorum* occur only in mountains at the western margins of the Neovolcanic Axis and the Mexican Plateau, respectively (Sites et al. 1992). More extreme examples are highlighted by *S. anahuacus* and *S. palaciosi*, each of which is endemic to a few volcanic peaks near the Valley of México (Sites et al. 1992). Under a traditional taxonomic model, *S. grammicus* has the most extensive distribution of any member of the group, ranging from the southern end of the Mexican Plateau to the lower Rio Grande Valley of southern Texas (Sites et al. 1992). One potential complication is that the taxonomic status of populations in this species is presently in flux (Marshall et al. 2006), and it has been suggested that at least two subspecies (*S. g. disparilis* and *S. g. microlepidotus*) may warrant recognition as separate species (Lara-Góngora 2004). As a consensus on the relationships of the various subspecies of *S. grammicus* has apparently not emerged, we retain recognition of the species as defined by Sites et al. (1992).

Partly as a consequence of the broad geographic distribution of *S. grammicus* (*sensu* Sites et al. 1992), the investigation of life-history traits in general, and patterns of reproductive activity in particular, have been productive bases of investigation (see Ramírez-Bautista et al. 2004; Hernández-Salinas et al. 2010 and references therein). Notably, most of these previously studied populations exhibit the same basic pattern, reproductive asynchrony; gametogenesis, courtship, and mating all occur in the summer or fall, pregnancy proceeds over winter, and parturition occurs the following early spring (e.g., Guillette and Casas-Andreu 1980; Jiménez-Cruz et al. 2005). In addition, the same general pattern has been documented in populations distributed across a wide elevational gradient (Ramírez-Bautista et al. 2004). Nevertheless, there are several reasons why we might expect geographic variation in reproductive traits of this species to be substantially more extensive than currently understood. First, *S. grammicus* occurs in a wide variety of habitats across a relatively large geographic area distinguished by substantial topographic and climatic complexity (Leyte-Manrique 2011). Second, molecular and karyotypic studies have revealed significant genetic variation in this species (Arévalo et al. 1991; Marshall et al. 2006). This variation appears to be most extensive among populations in the southern part of its range (i.e., central México), some of which may represent independent evolutionary lineages (e.g., Arévalo et al. 1991; Marshall et al. 2006; Leyte-Manrique 2011). Previous work has found evidence of differences between high- (>2700 m) and low-elevation (≤2700 m) populations of *S. grammicus* in a number of behavioral and physiological aspects of reproduction (e.g., the onset and duration of vitellogenesis, ovulation, and gestation) as well as morphological traits (e.g., body size at birth and sexual maturity; litter size: Ramírez-Bautista et al. 2004; Leyte-Manrique 2011). However, known variation in life-history traits in this species is nevertheless likely to be incomplete; given that reproductive traits can be influenced by both environmental and genetic factors, a lack of information from many areas expected to differ in these factors relative to previously studied populations constrains our ability to fully characterize the extent of life-history variation in *S. grammicus*. More broadly, studies of this species in particular can inform our understanding of life-history variation in other widely distributed species.

In this study, we address the following general questions with respect to reproduction in a low-elevation population of *S. grammicus*: (1) what is the extent of sexual dimorphism in this population? (2) What is the pattern of the annual reproductive cycle in males and females? (3) Is peak reproductive activity associated with environmental factors (temperature, precipitation, or photoperiod)? (4) Does litter size covary with female snout–vent length (SVL)? (5) How do the reproductive characteristics of *S. grammicus* from the low-elevation site we studied compare with those observed in other populations of this species?

## Materials and Methods

### Study species

**Figure 1:**
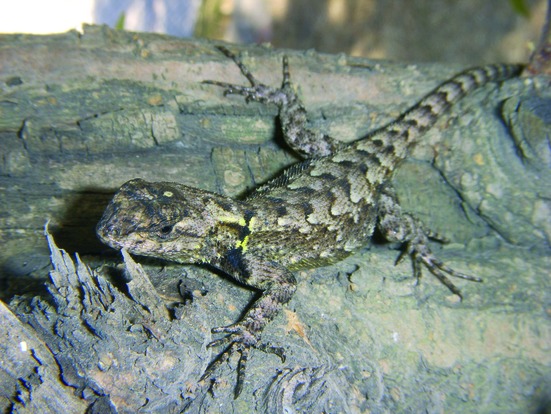
Adult female of *Sceloporus grammicus* from Tepeapulco, Hidalgo, México.

**Figure 2:**
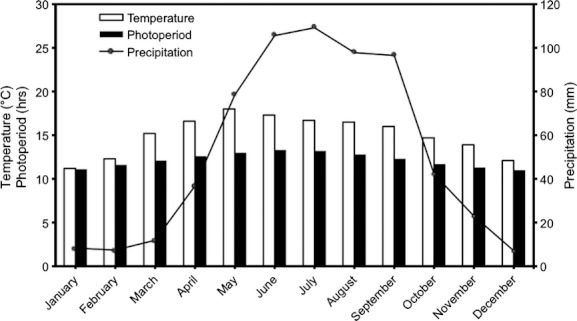
Monthly temperature, precipitation, and photoperiod data for Tepeapulco, Hidalgo, México. Temperature and precipitation data from García (1981); photoperiod data from the Astronomical Almanac (1984).

*Sceloporus grammicus* is a small (≤65 mm SVL) viviparous spiny lizard common in a variety of habitats throughout central México (Fig. 1; see Introduction). We studied a population of *S. grammicus* near the town of Tepeapulco, Hidalgo, México (19°47′N, 98°33′W), located at an elevation of 2578 m. Mean annual temperature at the study site is 15°C, and the average annual rainfall is 624 mm. The dominant vegetation is scrub crasicaule, which is dominated by nopal (*Opuntia* sp.), huizache (*Acacia* sp.), mesquite (*Mimosa buncifera*), and yucca (*Yucca filifera*, *Y. decipiens*; Rzedowski 1978). Temperature and precipitation data collected over a 23-year period were taken from García (1981), and the Astronomical Almanac (1984) provided data on photoperiod. These data were used to construct a climatic profile for the region representative of the study area (Fig. 2).

### Morphological and gonadal measurements

Between July 2003 and June 2004, we collected a total of 126 individuals, representing 57 adult males, 52 adult females, 6 juveniles, and 11 neonates. Following the collection of lizards in the field, external morphological data on lizards were obtained (see below). Lizards were anesthetized in the laboratory by lowering body temperature, and quickly killed by injection of a dose of 10% formalin behind the skull on the neck. Specimens were fixed in 10% formalin (Ramírez-Bautista et al. 2002, 2008a) and deposited in the vertebrate collections of the laboratory where gonadal analyses were subsequently performed (Population Ecology, Centro de Investigaciones Biológicas, Universidad Autónoma del Estado de Hidalgo, Pachuca, Hidalgo, México). The following linear measurements were recorded to ±1 mm on adult lizards: SVL, head length (HL), head width (HW), femur length (FL), forearm length (FOL), tibia length (TL), length and width of left testis in males, and length and width of left and right vitellogenic follicles, nonvitellogenic follicles, and freshly ovulated eggs (or embryos) in females (Ramírez-Bautista et al. 2008a; Hernández-Salinas et al. 2010). In addition, the number of ovarian follicles (nonvitellogenic and vitellogenic) and oviductal embryos was recorded. Vitellogenic follicles were distinguished from nonvitellogenic follicles by their relatively large size and presence of yellowish yolk, whereas nonvitellogenic follicles were smaller and translucent in appearance (Goldberg 1970). The smallest females containing vitellogenic follicles or embryos in the uterus were used to estimate minimum size at sexual maturity. Males were considered sexually mature if they showed enlarged testes, and enlarged and highly convoluted epididymides (Goldberg and Lowe 1966). Neonates and juveniles composed two subadult size classes (i.e., lizards that were not sexually mature as defined above) and were distinguished from each other by the much smaller SVL of neonates relative to juveniles. Livers and fat bodies were removed and weighed to ±0.0001 g on a balance. Litter size was determined by counting the number of embryos in the oviducts of adult females during the reproductive season. We determined the stages of embryonic development according to Dufaure and Hubert (1961).

### Statistics

Analyses of morphological and gonadal traits were restricted to sexually mature males and females. For tests of sexual dimorphism, we removed an effect of body size (SVL) using linear regression and then used the residuals in statistical tests. We also tested for differences across months in several morphological traits associated with reproductive development (gonadal mass, liver mass, fat body mass). As data did not meet conditions for parametric tests, we used nonparametric approaches for hypothesis testing. We used multiple regressions to test for a correlation of three environmental parameters with male and female gonadal mass. Simple regression was used to test for a relationship between litter size and female body size. Relative litter mass (RLM) was calculated as litter mass/(female mass − litter mass; Vitt and Congdon 1978). We used the sequential Bonferroni adjustment (Rice 1989) to control α at 0.05 when performing multiple tests of the same hypothesis. Means are presented ±SE unless otherwise indicated. All statistical analyses were performed with the MYSTAT version of SYSTAT 12 (SYSTAT Software, Inc., Chicago, IL, USA).

## Results

### Sexual size dimorphism

Sexually mature males of *S. grammicus* ranged from 43 to 75 mm SVL (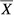 = 59 ± 1 mm, *n* = 57), whereas sexually mature females ranged from 42 to 66 mm SVL (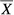 = 55 ± 1 mm, *n* = 52). Males were larger than females (Mann–Whitney *U* = 1052, *P* = 0.009); had longer and wider heads relative to their body size (HL: Mann–Whitney *U* = 836.5, *P* < 0.001; HW: Mann–Whitney *U* = 853, *P* < 0.001); and had proportionately longer forelimbs (Mann–Whitney *U* = 1156.5, *P* = 0.048), femurs (Mann–Whitney *U* = 941.5, *P* = 0.001), and tibias (Mann–Whitney *U* = 769, *P* < 0.001; Table 1).

**Table 1:** Descriptive statistics of morphometric traits of male and female *Sceloporus grammicus* from Tepeapulco, Hidalgo, México

Trait	Males 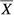	Females 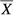
SVL (mm)	59 ± 1	55 ± 1
Mass (g)	6.9 ± 0.3	6.1 ± 0.3
Head width (mm)	10.2 ± 0.2	9.2 ± 0.1
Head length (mm)	13.0 ± 0.2	11.8 ± 0.1
Forearm length (mm)	8.0 ± 0.2	7.3 ± 0.1
Femur length (mm)	12.4 ± 0.2	11.1 ± 0.2
Tibia length (mm)	9.8 ± 0.2	8.5 ± 0.1

Means are given ±SE.

### Male reproductive cycles

**Figure 3:**
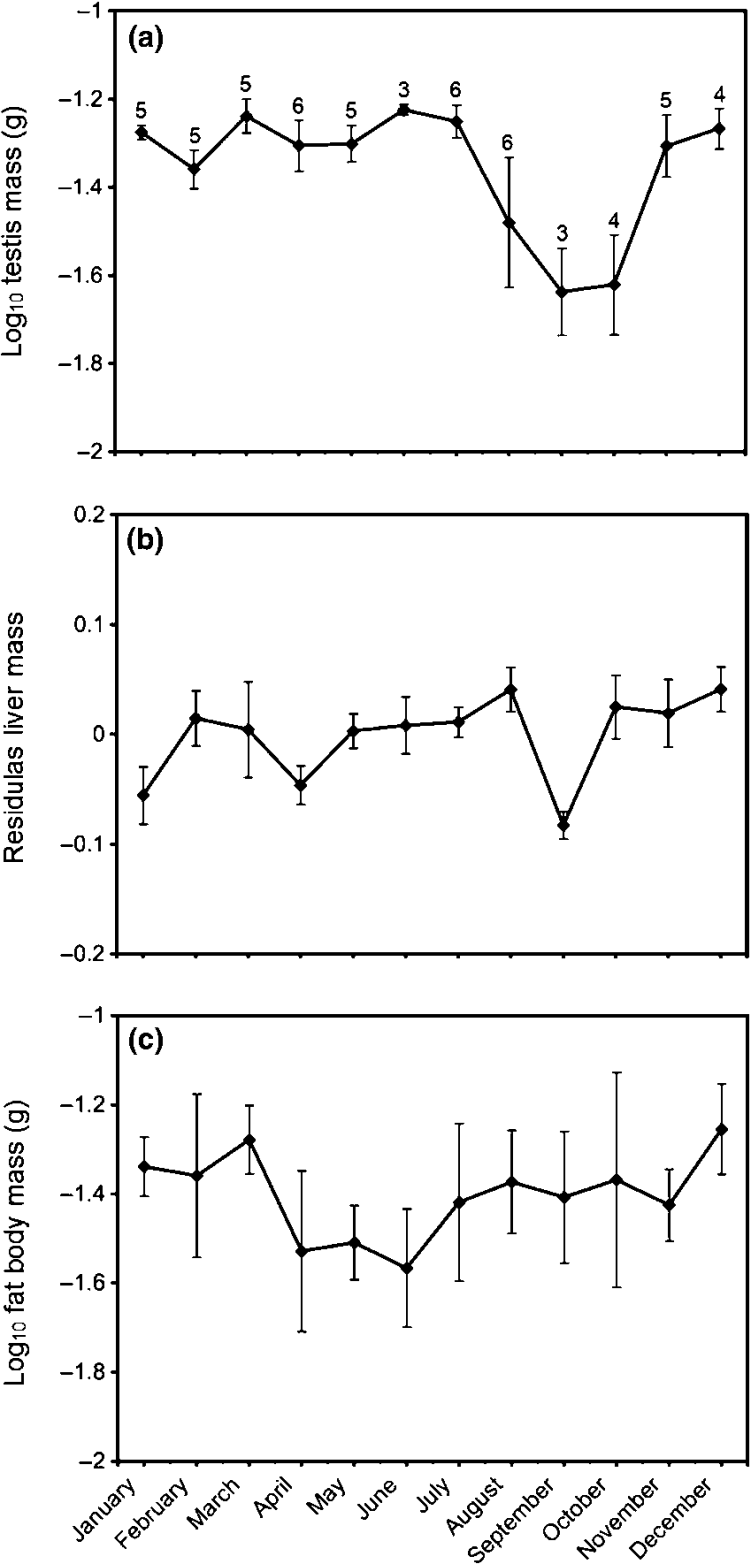
Monthly change in mass of (a) male gonads, (b) livers, and (c) fat bodies for *Sceloporus grammicus* at Tepeapulco. Values above error bars indicate monthly sample sizes.

There was no relationship between SVL and left testis mass (= testis mass; *F*_1,55_ = 1.9, *r*^2^ = 0.03, *P* = 0.18), or SVL and fat body mass (*F*_1,55_ = 0.4, *r*^2^ = 0.01, *P* = 0.54). However, there was a significant positive relationship between liver mass and SVL (*F*_1,55_ = 17.7, *r*^2^ = 0.24, *P* < 0.001). Thus, liver cycle is represented by a plot of regression residuals, in contrast to log-transformed testis mass and fat body mass (Fig. 3a–c). There was no significant difference in unadjusted testis mass or fat body mass as a function of month (testis mass: Kruskal–Wallis *H* = 18.6, *P* = 0.07, Fig. 3a; fat body mass: Kruskal–Wallis *H* = 9.6, *P* = 0.57, Fig. 3c). There was a trend toward a difference in size-adjusted liver mass across months, but this difference was not significant following sequential Bonferroni adjustment (Kruskal–Wallis *H* = 19.8, *P* = 0.048, Fig. 3b). Male testes began to enlarge in October and November and reached maximum size in June (Fig. 3a). Multiple regression revealed no significant relationship between testis mass and three environmental variables (overall model: *F*_3,8_ = 1.7, *r*^2^ = 0.39, *P* = 0.24; temperature: *P* = 0.28; precipitation: *P* = 0.10; photoperiod: *P* = 0.07).

### Female reproductive cycles

**Figure 4:**
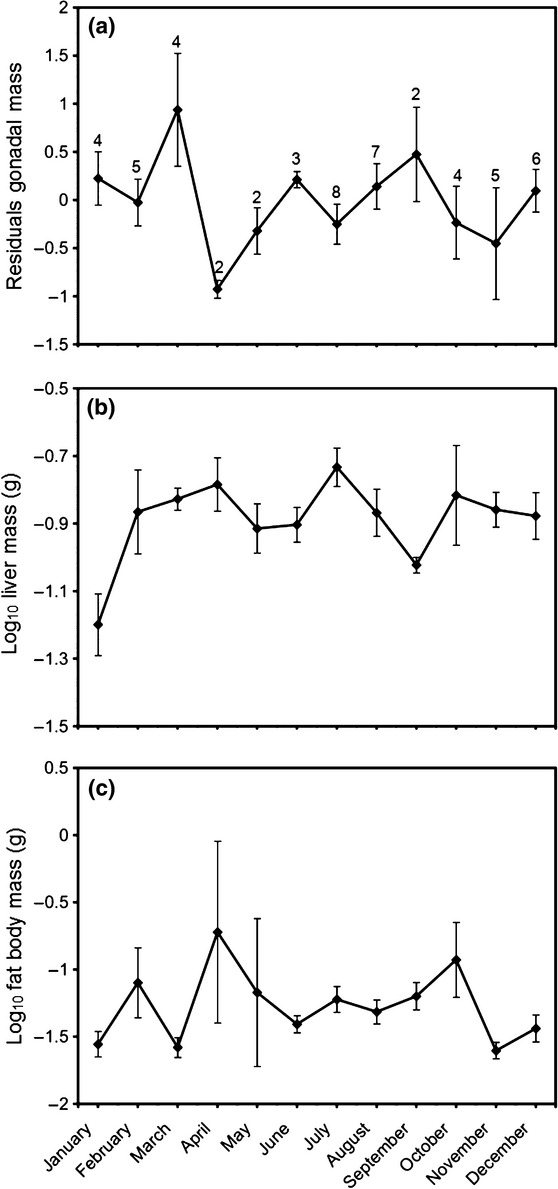
Monthly change in mass of (a) female gonads, (b) livers, and (c) fat bodies for *Sceloporus grammicus* at Tepeapulco. Values above error bars indicate monthly sample sizes.

Female SVL was significantly correlated with gonadal mass (*F*_1,50_ = 27.4, *r*^2^ = 0.35, *P* < 0.001), but not fat body mass (*F*_1,50_ = 0.5, *r*^2^ = 0.01, *P* = 0.46) or liver mass (*F*_1,50_ = 0.5, *r*^2^ = 0.01, *P* = 0.49). We removed the effects of female size by using the regression residuals to describe the gonadal cycle, but the fat body and liver cycles are represented by log-transformed organ mass (Fig. 4a–c). There was no significant difference across months in size-adjusted gonadal mass (Kruskal–Wallis *H* = 12.8, *P* = 0.31, Fig. 4a), or unadjusted liver mass (Kruskal–Wallis *H* = 14.5, *P* = 0.21, Fig. 4b) or fat body mass (Kruskal–Wallis *H* = 14.8, *P* = 0.19, Fig. 4c). Female gonadal mass was not correlated with any environmental parameter in a multiple regression analysis (overall model: *F*_3,8_ = 0.3, *r*^2^ = 0.09, *P* = 0.85; temperature: *P* = 0.71; precipitation: *P* = 0.67; photoperiod: *P* = 0.89).

Females with vitellogenic follicles were observed in October (2/4 females, 50%), November (2/5 females, 40%), December (1/6 females, 17%), January (2/4 females, 50%), February (1/5 females, 20%), April (1/2 females, 50%), May (2/2 females, 100%), and July (3/8 females, 38%). Embryonic development was observed in December (stages 25–35; 5/6 females, 83%), January (stages 35–38; 2/4 females, 50%), February (stage 35; 2/5 females, 40%), March (stages 39–40; 4/4 females, 100%), April (stage 35; 2/2 females, 100%), June (stages 38–40; 2/3 females, 67%), July (stages 35–40; 2/8 females, 25%), August (stages 38–40; 6/7 females, 86%), September (stage 40; 2/2, 100%), October (stage 35; 1/4 females, 25%), and November (stage 35; 1/5 females, 20%). Thus, the female population exhibits embryos through the year. However, neonates (*n* = 11) were collected in the field only from May to October and juveniles (*n* = 6) from April to September.

The number of vitellogenic follicles (8.8 ± 0.9, range: 3–17, *n* = 14) differed significantly from the number of embryos (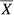 = 5.1 ± 0.3, range: 2–8, *n* = 27; Mann–Whitney *U* = 57.5, *P* < 0.001); thus, the number of embryos was used to estimate litter size. Litter size was not significantly correlated with SVL (*F*_1,25_ = 0.1, *r*^2^ < 0.01, *P* = 0.74). Mean RLM was 0.35 ± 0.04 embryos (range = 0.11–1.02, *n* = 27); RLM was not correlated with female SVL (*F*_1,25_ = 1.04; *r*^2^ = 0.04, *P* = 0.32). There was also no significant variation in RLM across months (Kruskal–Wallis *H* = 11.2, *P* = 0.26). Size at birth in *S. grammicus* from Tepeapulco ranged from 25 to 34 mm SVL (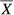 = 30.1 ± 0.9 mm, *n* = 11).

## Discussion

Asynchronous reproductive cycles are characteristic of multiple lineages of montane viviparous lizard species in central México, including Phrynosomatidae (Guillette 1982; Feria-Ortiz et al. 2001; Ramírez-Bautista et al. 2008a; Hernández-Salinas et al. 2010), Scincidae (Guillette 1983; Ramírez-Bautista et al. 1996, 1998), Anguidae (Guillette and Casas-Andreu 1987), and Xantusiidae (Ramírez-Bautista et al. 2008b). In such systems, mating occurs several months prior to ovulation, and sperm transfer is thus temporally uncoupled from fertilization. Typically, male testicular recrudescence is initiated in the spring. This corresponds to an increase in testicular mass and development of spermatogonia, followed by the subsequent transformation of these cells into primary spermatocytes, secondary spermatocytes, round and elongate spermatids, and ultimately mature spermatozoa (sperm). The asynchrony in male and female reproductive cycles arises because mating occurs immediately following mature sperm production in the late summer or early fall, several months in advance of female vitellogenesis, and mature egg production. Females store sperm in oviductal sacs (spermathecae) until ovulation and subsequent fertilization by the female. The evolution of reproductive asynchrony in high-elevation populations of Mexican *Sceloporus* is thought to be adaptive by allowing for parturition during the period of greatest food abundance (spring), in turn permitting rapid growth (and thus survival) in first-year offspring (Ramírez-Bautista 1995; Lemos-Espinal et al. 1998). At the proximate level, temporal differences in the onset of reproductive activity (i.e., reproductive asynchrony) in males and females can arise if the sexes use different environmental cues to initiate reproductive activity (Guillette 1983; Ramírez-Bautista et al. 2008a,b).

### Sexual dimorphism

We found evidence of substantial sexual size dimorphism in *S. grammicus* at Tepeapulco; males exhibited larger morphological characteristics than females in all traits compared (Table 1). Minimum size at sexual maturity was similar for males (43 mm) and females (42 mm). This indicates that observed differences in adult size between the sexes reflect differences in age-based patterns of mortality (Ruby and Dunham 1984), growth rates (Dunham 1982; Cox and John-Alder 2007), or both (Smith and Ballinger 1994; Cox 2006). Like most other species of *Sceloporus*, adult male *S. grammicus* expresses sexually dimorphic throat and abdominal patches. These features are ordinarily hidden and revealed only during social interactions, especially courtship displays and agonistic interactions with other adult males (Cooper and Greenberg 1992; Carpenter 1995; Ramírez-Bautista and Vitt 1997). As in other phrynosomatids, sexual dimorphism in *S. grammicus* is likely maintained by sexual selection (Ruby 1978, 1981). In lizards, sexual selection is usually linked to male–male competition (e.g., Olsson 1994), though there is some evidence for female choice, both overt (Bleay and Sinervo 2007) and cryptic (Calsbeek and Sinervo 2004). Either mechanism would provide a logical basis for the observed differences in morphological traits seen in this study.

Finally, we note that males (59.0 mm) and females (55.0 mm) of *S. grammicus* from Tepeapulco are larger (SVL) than those at Teotihuacan (males: 57.0 mm; females: 55.0 mm; Jiménez-Cruz et al. 2005) and Pachuca (males: 54.0 mm; females: 52.0 mm; Ramírez-Bautista et al. 2005). Geographic variation in body size has been linked to local differences in elevation in some species (Michaud and Echternacht 1995; Ramírez-Bautista et al. 2004); however, Teotihuacan, Pachuca, and Tepeapulco occur at similar elevation (Table 2). The observed differences in body size among populations of *S. grammicus* may thus be an adaptive response to other environmental factors that differ between these three sites (Ramírez-Bautista et al. 2004), possibly indicative of the extent of reproductive isolation (e.g., Arévalo et al. 1991).

**Table 2:** Female reproductive characteristics in populations of *Sceloporus grammicus*

Population	Elevation (m)	Period vitellogenesis	Gestation	Period Parturition	Mating pattern	Mean Litter size	Range litter size	Mean SVL (mm)	Range SVL (mm)
Tepeapulco^1^	2578	October–July	December–November	May–October	S	5.1 ± 0.3	2–8	55 ± 1	42–66
Teotihuacan^2^	2294	October–November	November–April	February–April	A	5.1 ± 0.2	2–9	48.8 ± 0.6	44.1–72.3
Pachuca^3^	2435	July–November	November–May	May–July	A	5.2 ± 0.2	2–10	51.7 ± 0.5	40.0–67.0
Oaxaca^4^*	1950	August–November	November–April	April	A	4.9 ± 0.16	3–7	57.4 ± 0.9	45.0–68.0
S. Texas^5^*	–	July–September	November–May	May–June	S	5.4 ± 0.06	3–7	–	–
Pedregal San Angel^6^	2400	May–August	September–April	March	A	5.3 ± 0.19	2–11	53.04 ± 0.55	40.0–62.0
Cantimplora^6^	3300	May–July	October–May	April	A	3.7 ± 0.17	2–6	45.01 ± 0.32	34.0–55.0
Parq. Nac. Zoquiapan^7^	2000–3200	July–September	September–May	May–June	A	5.2 ± 0.25	3–7	48.5 ± 0.07	42.3–61.2
Michilia^8^*	2480	August–December	January–May	May	S	6.2 ± 1.7	3–9	–	44.0–60.0
Monte Alegre Ajusco^9^	3200	July–September	November–April	April–May	A	3.51 ± 0.16	2–6	48.8 ± 0.61	37.9–54
Capulin^9^	3400	July–September	November–April	April–May	A	3.72 ± 0.14	2–6	44.5 ± 0.6	38.6–50
La Estanzuela^10^	2700	May–November	November–April	March	A	4.4 ± 0.27	3–6	49.3 ± 0.63	41.6–57.4
Tilcuautla^10^	2472	June–November	November–March	March	A	5.6 ± 0.63	3–12	52 ± 0.63	44.6–62.4

A, asynchronous; S, synchronous; SVL, snout–vent length.

* Population extralimital to Central Plateau of México.

1 This study.

2 Jiménez-Cruz et al. (2005).

3 Ramírez-Bautista et al. (2005).

4 Ríos-Pérez (2005).

5 Guillette and Bearce (1986).

6 Martínez (1985).

7 Guillette and Casas-Andreu (1980).

8 Ortega and Barbault (1984).

9 Méndez-de la Cruz (1988).

10 Hernández-Salinas et al. (2010).

### Male reproductive cycles

Male testicular mass did not differ significantly across months (*P* = 0.07), indicating that male *S. grammicus* from Tepeapulco may be sexually responsive throughout the year. However, the nonsignificant effect of month on gonadal mass we found likely obscures a more complex picture. Inspection of Fig. 3A indicates that testicular mass is not uniform, but instead is greatest from November to July, and declines from August to October. This suggests that testicular recrudescence is initiated in mid-autumn, with maximum testicular size sustained through early summer, resulting in a lengthy reproductive period of about 9 months. Such a pattern of activity is unusual relative to that of other populations of *S. grammicus* (Guillette and Casas-Andreu 1980; Guillette and Bearce 1986; Jiménez-Cruz et al. 2005; Ramírez-Bautista et al. 2005), particularly with respect to duration and seasonality of male reproductive activity. For instance, males from two other study sites near the southern end of the distribution of *S. grammicus*, Teotihuacan and Pachuca, showed maximum reproductive activity over 4 months (July–October: Jiménez-Cruz et al. 2005) and 2 months (July–August: Ramírez-Bautista et al. 2005), as opposed to the 9-month, November–July pattern we found at Tepeapulco. Notably, none of the environmental factors that we examined (temperature, precipitation, and photoperiod) were correlated with testicular activity in males from Tepeapulco, contrary to that observed in many other lizard species (Marion 1982; Ramírez-Bautista et al. 1995; Ramírez-Bautista and Vitt 1998). The unusual pattern of male reproductive activity observed in this population explains why it is not correlated with the environmental factors we considered (Fig. 3).

### Female reproductive cycles

Females from Tepeapulco were found with either vitellogenic follicles or embryos in all months of the year (Table 2). Embryos in advanced stages of development were found in January–April (stages 39–40) and June–December (stages 25–40). Parturition appears to occur from March or April to October, although we did not encounter neonates in the field until May (Table 2). Female reproductive activity in Tepeapulco exhibits a markedly different pattern from that observed in all previously studied populations of *S. grammicus*. In these earlier studies, vitellogenesis was generally found to be restricted to the wet season (July–November), followed by ovulation and fertilization in November and December, embryonic development during the dry season (winter), and parturition in the spring (Guillette and Bearce 1986; Ramírez-Bautista et al. 2005; Jiménez-Cruz et al. 2005; Hernández-Salinas et al. 2010; Table 2). This latter pattern is characteristic of most live-bearing high-elevation *Sceloporus* species that inhabit the temperate regions (e.g., *S. jarrovii*: Goldberg 1970, 1971; Ballinger 1973; Ramírez-Bautista et al. 2002; *S. grammicus microlepidotus*: Guillette and Casas-Andreu 1980; *S. bicanthalis*: Guillette 1982; *S. torquatus*: Guillette and Méndez-de la Cruz 1993; Feria-Ortiz et al. 2001). Fall reproductive activity is also characteristic of some lowland lizard species (e.g., *Gerrhonotus liocephalus*: Flury 1949; *Eumeces egregious*: Mount 1963; *S. poinsettii*: Ballinger 1973; Gadsden et al. 2005; *S. grammicus disparilis*: Guillette and Bearce 1986). Notably, all these species have asynchronous reproduction, except *S. grammicus disparilis*. In most populations of *S. grammicus* from high elevation, parturition occurs during a short period, typically from March to May (e.g., Guillette and Casas-Andreu 1980). In contrast, parturition in lowland populations is extended, from March to as late as July (e.g., Ramírez-Bautista et al. 2005; Hernández-Salinas et al. 2010), or from May to October (this study). As with males, there was no correlation of female reproductive cycling with any of three environmental parameters (temperature, precipitation, and photoperiod) linked to female reproductive cycles in some other lizard species (Marion 1982; Ramírez-Bautista and Vitt 1997, 1998).

Litter size in *S. grammicus* from Tepeapulco is consistent with that observed in other populations of this species (see Table 2). Populations from high elevation have smaller litters relative to low-elevation populations (Martínez 1985; Méndez-de la Cruz 1988; Ramírez-Bautista et al. 2004, 2011). This is consistent with the idea that females from Tepeapulco are responding in a similar way to that observed in females from other low-elevation populations (Table 2), perhaps reflecting the presence of common abiotic environmental factors. Differences in litter size between high and low populations of *S. grammicus* could be a plastic response to local environmental conditions (reaction norms: Stearns 1993) or demographic pressures (Ballinger 1977; Dunham 1982). However, it could also be indicative of local adaptation; such an interpretation is consistent with the findings of Arévalo et al. (1991), who identified seven distinct chromosomal races of *S. grammicus* in Hidalgo state alone.

Relative litter mass in females from Tepeapulco increased with successive stages of embryonic development. Immediately following ovulation, RLM ranged from 0.22 to 0.26 (April and August, respectively), and prior to birth was 0.41 (January), 0.55 (March), and 0.80 (November). This pattern is similar to that observed in other populations of *S. grammicus* (Jiménez-Cruz et al. 2005), as well as other montane viviparous congeners such as *S. jarrovii* (Parker 1973; Ramírez-Bautista et al. 2002). These results also broadly correspond to the findings of Vitt and Price (1982) who found that RLM was higher among sit-and-wait lizards like *Sceloporus* than in actively foraging species such as the whiptail lizards of the genus *Aspidoscelis*.

### Summary and future directions

In contrast to most other populations of *S. grammicus*, and uniquely among those studied in the Central Plateau, we found that reproduction in males and females from Tepeapulco was synchronous, and unusually lengthy in duration (Figs. 3A, 4A). The basis for this shift is presently unknown, but could serve as an important starting point for future research. For example, are these population differences the result of a general plastic response to variable environmental conditions in this species, or is there a local heritable component to the timing and duration of reproductive activity? Did the evolution of an extended reproductive period in one sex helped drive an extended reproductive period in the other, or was the reproductive period in one or both sexes extended in response to selection from additional external factors? Such questions could be addressed using a combination of common garden rearing experiments in conjunction with the establishment of a robust phylogeny for the species on to which patterns of reproductive cycling could be mapped.

The advantages to males in a lengthy breeding season seem intuitive; namely, that additional mating opportunities could result. But what might be the benefit to females to having a lengthy breeding season? In oviparous lizards, an extended breeding season can allow females to produce multiple clutches per annum (Benabib 1994; Ramírez-Bautista et al. 1995, 2006). Viviparous species, on the other hand, produce only one clutch per year (Guillette 1982, 1983; Ramírez-Bautista et al. 2008a,b). One possible benefit is that female sperm storage may not be necessary in Tepeapulco, as has been hypothesized for other synchronously breeding populations of *S. grammicus* (Ortega and Barbault 1984) and *S. grammicus disparilis* (Guillette and Bearce 1986). This is in contrast with that interpreted for other populations of *S. grammicus* that exhibit asynchronous reproduction (e.g., Guillette and Casas-Andreu 1980; Jiménez-Cruz et al. 2005; Ramírez-Bautista et al. 2005; Hernández-Salinas et al. 2010). Presumably, females incur some cost in the maintenance of viable sperm in the reproductive tract (Cuellar 1966), either through investment in specialized structures to store sperm, production of nutrients to maintain their viability until fertilization, or both. If males from Tepeapulco can produce sperm through all or most of the year, mating (and thus, fertilization) could be accomplished when conditions for doing so are optimal for females without needing to invest in these additional and potentially costly morphological and physiological traits. Alternatively, if local conditions (e.g., temperature, food availability) are favorable for females to give birth throughout the year, this could help prevent the concentration of reproductive output from all females during a narrow time frame, possibly helping minimize the risk of predation to any one clutch. A similar response to predation is thought to partly explain the occurrence of multiple clutches in some tropical oviparous species (Benabib 1994; Ramírez-Bautista et al. 2006). Further investigation of the ecology and evolution of life-history strategies in the *S. grammicus* complex should be revealing with respect to these issues.
